# Metformin use is associated with a reduced risk of cognitive impairment in adults with diabetes mellitus: A systematic review and meta-analysis

**DOI:** 10.3389/fnins.2022.984559

**Published:** 2022-08-25

**Authors:** Jia-Hao Zhang, Xin-Yang Zhang, Yan-Qiu Sun, Ren-Hua Lv, Mei Chen, Meng Li

**Affiliations:** ^1^Laboratory of Laser Sports Medicine, School of Sports Science, South China Normal University, Guangzhou, China; ^2^Department of Rehabilitation Medicine, Guangdong Women and Children Hospital, Guangzhou, China; ^3^Department of Rehabilitation Medicine, Xiangtan Central Hospital, Xiangtan, China

**Keywords:** metformin, dementia, Alzheimer's disease, diabetes mellitus, cognitive dysfunction

## Abstract

**Objective:**

Controversy exists regarding the impact of metformin and whether it prevents or promotes the incidence of cognitive dysfunction. This systematic review and meta-analysis were conducted to identify the effect of metformin therapy on cognitive function in patients with diabetes.

**Methods:**

Electronic databases (PubMed, EMBASE, PsycINFO, the Cochrane Library, and Web of Science) were systematically searched by two investigators from the date of inception until March 1, 2022. The study followed PRISMA guidelines. Inclusion criteria were defined according to the ***PECOS*** model. Eligible studies investigated cognitive dysfunction in metformin users compared with non-users in adults with diabetes. Only observational study designs (such as cohort, cross-section, and case-control) were included.

**Results:**

A systematic search identified 1,839 articles, of which 28 (17 cohort, 8 case-control, and 3 cross-sectional studies) were included in the meta-analysis. Metformin reduced the occurrence of cognitive impairment in patients with diabetes [unadjusted hazard ratio (*HR*) = 0.67, 95% *CI*: 0.62–0.73; adjusted hazard ratio (a*HR*) = 0.92, 95% *CI*: 0.85–0.99]. In addition, the use of metformin was associated with a decreased risk of dementia (*HR* = 0.64, 95% *CI*: 0.59–0.69; *aHR* = 0.90, 95% *CI*: 0.84–0.96), while a random-effects meta-analysis indicated no significant effect of metformin on the risk of Alzheimer's disease (AD) (*HR* = 0.85, 95% *CI*: 0.60–1.22; *aHR* = 1.10, 95% *CI*: 0.95–1.28).

**Conclusion:**

Metformin therapy decreased the occurrence risk of cognitive decline in patients with diabetes mellitus. Moreover, the use of metformin by adults with diabetes for the prevention of dementia, but not AD, is supported by the available evidence.

## Introduction

Cognitive dysfunction, which includes delirium, mild cognitive deficits, and dementia, is characterized by a significant decline from a previously attained cognitive functional level (Sachdev et al., 2014; Zhang et al., 2020). Numerous epidemiological studies have increasingly recognized cognitive impairment as important comorbidity and complication of diabetes and it has become a major public health concern (Gispen and Biessels, 2000; Biessels and Despa, 2018; Biessels and Whitmer, 2020). A systematic review reported that patients with diabetes have a 73% increase in the risk of dementia and a 56% increase in the risk of Alzheimer's disease (AD) (Diniz Pereira et al., 2021). Moreover, the etiology of cognitive impairment in patients with diabetes is potentially multifactorial (Campbell et al., 2018; Jash et al., 2020; Yuan et al., 2021). For example, poor glycemic control and the presence of microvascular complications, such as neuropathy and retinopathy, have also been associated with cognitive dysfunction (Moheet et al., 2015); insulin resistance also may increase the occurrence risk of AD (Baker et al., 2011; Lyu et al., 2020).

Metformin is a primary oral hypoglycemic agent widely used for treating diabetes since 1950s (Flory and Lipska, 2019). Metformin functions predominantly by improving the sensitivity of insulin receptors to insulin, which enhances glucose uptake and decreases hepatic glycogen synthesis at low glucose (Hundal et al., 2000; Satoh, 2014). However, the function of metformin is not confined to glucose reduction (Liu et al., 2014). Increasing evidence has emerged indicating that metformin can penetrate the blood-brain barrier to improve cerebral energy metabolism in some regions of the brain associated with semantic memory and some white matter in adults with diabetes (Huang et al., 2014; Sritawan et al., 2020). Moreover, an animal experiment supported the fact that metformin treatment prevents amyloid plaque deposition and reduces memory impairment (Ou et al., 2018).

Several studies have reported that metformin could negatively impact cognitive function (Hsiao et al., 2014; Ha et al., 2021). For example, Chen et al. (2009) found that the activation of AMP-activated protein kinase (AMPK) by metformin raised the production of β-secretase to promote the deposition of β-amyloid peptides (Aβ), which leads to cognitive dysfunction (Chen et al., 2009). In addition, a case-control study of patients aged 65 years or older indicated that the long-term metformin use increased the risk of AD [(*OR*): 1.71, 95% *CI:* 1.12–2.60] (Imfeld et al., 2012). Therefore, the effect of metformin and whether it is associated with the prevention or promotion of the incidence of cognitive impairment is controversial.

This meta-analysis aimed to analyze the available evidence on the use of metformin and cognitive function in adults with diabetes and ascertain the relationship between the two.

## Methods

### Search strategy

The databases (PubMed, EMBASE, PsycINFO, Cochrane Library, and Web of Science) were screened independently by two investigators (JHZ and YQS) from their inception date until March 1, 2022. The search strategy for the PubMed database is presented in Table 1. In addition, reference lists from identified and relevant reviews were manually searched.

**Table 1:** Search strategy of PubMed database.

**Search**	**Query**
#1	“Cognition disorders”[MeSH Major Topic] OR “disorder cognition”[Title/Abstract] OR “disorders cognition”[Title/Abstract] OR “memory”[MeSH Major Topic] OR “mental recall”[MeSH Major Topic] OR “Dementia”[MeSH Major Topic] OR “Dementias”[Title/Abstract] OR “Amentia”[Title/Abstract] OR “Amentias”[Title/Abstract] OR “Senile”[Title/Abstract] OR “seniles”[Title/Abstract] OR “senility”[Title/Abstract] OR “paranoid dementia”[Title/Abstract] OR “dementias”[Title/Abstract] OR “senile paranoid”[Title/Abstract] OR “Paranoid”[Title/Abstract] OR “paranoids”[Title/Abstract] OR “dementia senile”[Title/Abstract] OR “Paranoid”[ Title/Abstract] OR “paranoids”[ Title/Abstract] OR “familial dementia”[Title/Abstract] OR “dementia familial”[Title/Abstract] OR “dementias familial”[Title/Abstract] OR “familial dementias”[Title/Abstract] OR “cognition”[MeSH Major Topic] OR “cognitions”[Title/Abstract] OR “cognitive function”[Title/Abstract] OR “cognitive functions”[Title/Abstract] OR “function cognitive”[Title/Abstract] OR “functions cognitive”[Title/Abstract] OR “alzheimer disease”[MeSH Major Topic] OR “Mini mental state examination”[Title/Abstract]
#2	“Metformin”[MeSH Major Topic] OR “Dimethylbiguanidine”[Title/Abstract] OR “Dimethylguanylguanidine”[Title/Abstract] OR “Glucophage”[Title/Abstract] OR “metformin hydrochloride”[Title/Abstract] OR “hydrochloride metformin”[Title/Abstract] OR “metformin hcl”[Title/Abstract]
#3	#1 AND #2

### Selection criteria

This systematic review and meta-analysis were conducted based on the preferred reporting items for systematic reviews and meta-analyses (PRISMA) guidelines (Moher et al., 2009). The protocol for this systematic review and meta-analysis was registered at INPLASY (registration number: INPLASY202250065). Studies were only selected for inclusion in accordance with the following ***PECOS*** criteria. ***P***articipants: all patients are individuals with diabetes aged 18 years or older and have no history of cognitive disorder. ***E***xposure: taking metformin monotherapy at any dosage for any duration. ***C***omparator: participants received other antidiabetic drugs rather than metformin or no therapy as the control group. ***O***utcomes: studies that investigated the risk (or incidence) of cognitive dysfunction were eligible for inclusion. ***S***tudy: only published observational study designs—such as cohort, case-control, or cross-sectional studies—were eligible for inclusion. In addition, detailed meeting summary information was included. Studies of randomized controlled trials (RCTs), case reports/series, basic science, and reviews were excluded.

### Data extraction and quality assessment of each study

Data from all eligible studies were extracted onto a standardized Excel spreadsheet independently by two investigators (JHZ and YQS). The following data were abstracted from each included study: publication details (such as first author and year of publication), study design, number of participants, participant characteristics (mean age and age range), gender, comparator, exposure, number of events, years enrolled, and outcomes (diagnosis and diagnostic criteria). For any discrepancies, a consensus was reached *via* discussion between the two investigators; if any uncertainty remained regarding inclusion, a senior author (XYZ) was consulted. To acquire relevant missing data from included studies, the first and/or corresponding authors of the studies were contacted.

Subsequently, JHZ and YQS independently evaluated the quality of each included study. The Newcastle–Ottawa Quality Assessment Scale (NOS) was used for cohort studies and case-control studies (Stang, 2010), while cross-sectional studies were appraised by the Agency for Healthcare Research and Quality (AHRQ) (Li et al., 2020).

### Data synthesis

The Review Manager software (version 5.4) was used to conduct the meta-analysis and sensitivity analysis, and publication bias was performed by STATA software (version 15.0). In accordance with the study of Jatho et al. (2021), the odds ratio (*OR*), relative risk (*RR*), or hazard ratio (*HR*) with a 95% confidence interval (*CI*) were selected as the effect size for included studies. Adjusted *OR*/*HR*/*RR* (accounting for confounding variables) and unadjusted *OR*/*HR*/*RR* were conducted. Heterogeneity was assessed using Higgins I-squared (*I*^2^) (*I*^2^ > 50% was regarded as significant heterogeneity) (Higgins et al., 2003). Publication bias was examined by Begg's funnel plot and Egger's test (Macaskill et al., 2001). Publication bias is present if Begg's funnel plot shows asymmetry or the *p*-value of Egger's test is less than 0.05. A sensitivity analysis was conducted by moving each study individually. All statistical significance was set at *p* < 0.05 (two-tailed).

Potential sources of heterogeneity were explored by conducting a subgroup analysis after sensitivity analysis. The following categorical variables were examined in the subgroup analysis: (1) dementia: oral metformin vs. oral other hypoglycemic drugs rather than metformin in patients with diabetes; (2) Alzheimer's disease: oral metformin vs. oral other hypoglycemic drugs rather than metformin in patients with diabetes.

## Results

### Study selection

**Figure 1:**
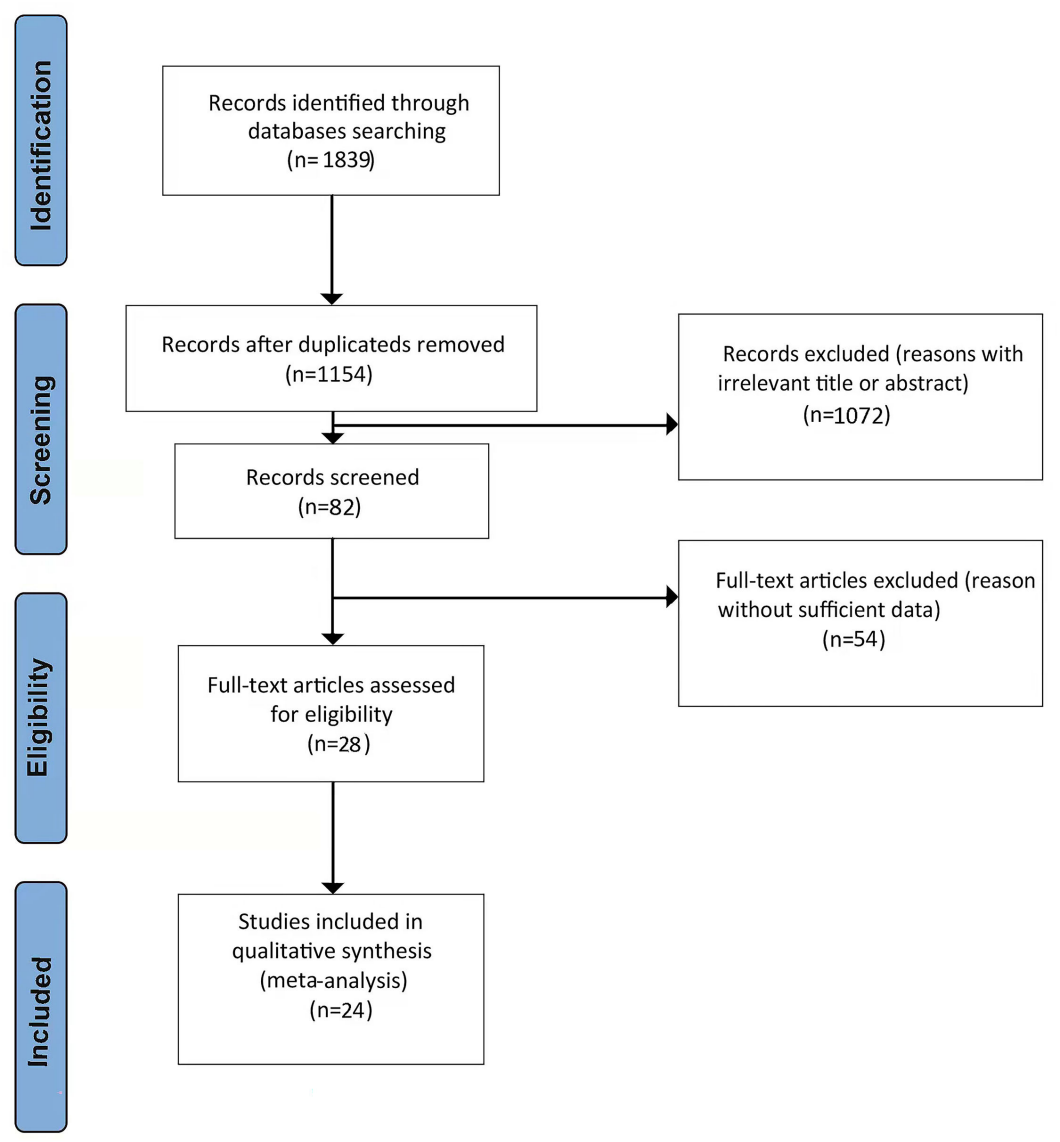
PRISMA flow diagram.

Figure 1 shows a PRISMA flow diagram of the literature search. A total of 1,839 potentially relevant studies were initially identified from the literature search. Of these, 685 duplicate articles that had been retrieved through electronic databases were removed. Another 1,072 irrelevant references were discarded after screening the titles and abstracts, and a further 54 studies were excluded for having insufficient data. Finally, 28 studies (Hsu et al., 2011; Imfeld et al., 2012; Moore et al., 2013; Whitmer et al., 2013; Cheng et al., 2014; Hsiao et al., 2014; Ng et al., 2014; Yokoyama et al., 2015; Liccini et al., 2016; Naharci et al., 2016; Orkaby et al., 2017; Bohlken et al., 2018; Kim et al., 2019; Koo et al., 2019; Porter et al., 2019; Scherrer et al., 2019a,b; Shi et al., 2019; Tseng, 2019; Weinstein et al., 2019; Wium-Andersen et al., 2019; Akimoto et al., 2020; Salas et al., 2020; Secnik et al., 2020; Sluggett et al., 2020; Ha et al., 2021; Sečník, 2021; Teng et al., 2021) remained and 24 studies were deemed appropriate in the pooled analysis after the screening of the initial 1,839 articles.

### Sample characteristics

The characteristics of the 28 included studies on the occurrence risk of cognitive dysfunction in diabetes with oral metformin are shown in Table 2. The 28 studies comprised seventeen cohort studies (Hsu et al., 2011; Moore et al., 2013; Whitmer et al., 2013; Cheng et al., 2014; Naharci et al., 2016; Orkaby et al., 2017; Kim et al., 2019; Koo et al., 2019; Porter et al., 2019; Scherrer et al., 2019a,b; Shi et al., 2019; Tseng, 2019; Weinstein et al., 2019; Salas et al., 2020; Secnik et al., 2020; Sečník, 2021), eight case-control studies (Imfeld et al., 2012; Hsiao et al., 2014; Bohlken et al., 2018; Wium-Andersen et al., 2019; Akimoto et al., 2020; Sluggett et al., 2020; Ha et al., 2021; Teng et al., 2021), and three cross-sectional studies (Ng et al., 2014; Yokoyama et al., 2015; Liccini et al., 2016). The articles were all published between 2012 and 2021, and their enrolment periods ranged from 1995 to 2019 except for two articles (Moore et al., 2013; Naharci et al., 2016) for which the enrollment periods were not reported. Sample sizes varied from 278,290 (Kim et al., 2019) to 193 (Naharci et al., 2016). Additional details on the covariates that were adjusted for in the statistical analyses are included in Supplementary Table 1.

**Table 2:** Summary of characteristics of the included studies.

**Study**	**Study design**	**N**	**Age^a^ yrs (range)**	**Gender: Male (%)**	**Comparator**	**Exposure**	***N*/events**	**Years enrolled**	**Outcome(s) - Diagnosis - Diagnostic criteria**
Ha et al. (2021)	Nested case-control study	70,499	NR (≥50)	43.6%	Non-use of metformin	Metformin-use	Metformin = 8,972/NR; No-metformin = 1,130/NR	2002–2017	- AD diagnosis - ICD-10
Sluggett et al. (2020)	Nested case-control study	29,412	80.6 (76.3–84.4)	40.2%	Non-use of metformin	Metformin-use	Metformin = 21,753/7,225; No-metformin = 5,464/1,839	2005–2011	- AD diagnosis - NINCDS-ADRDA and DSM-IV
Imfeld et al. (2012)	Case-control study	14,172	80.7 (≥65)	31%	Non-use of metformin	Metformin-use	Metformin = 147/76; No-metformin = 13,538/ 6,802	1998–2008	- AD diagnosis - At least two recordings of an AD diagnosis (a specific dementia test, a referral to a specialist, an assessment based on a neuroimaging technique; or an AD diagnosis preceded or followed by any recorded dementia symptoms)
Hsiao et al. (2014)	Case-control study	65,620	NR	NR	Non-use of metformin	Metformin-use	Overall (metformin or other oral hypoglycemic agents) = 65,620/462	1999–2011	- AD diagnosis - NR
Orkaby et al. (2017)	Retrospective cohort study	28,640	75.3 (≥65)	98.9%	Sulfonylureas-use	Metformin-use	Metformin = 17,200/3,202; Sulfonylureas = 11,440/4,211	2001–2012	- Dementia diagnosis - ICD-9 (290.x, 291.2, 294.1, 294.11, 331.x (except 331.83 [MCI]), 333.0, 333.4, 797, 332.0, 294.8, 046.1, and 046.3)
Weinstein et al. (2019)	Cohort study	12,044	67.9 (NR)	50.5%	Non-use of metformin	Metformin-use	Overall (dementia) = 3,315/349; Overall (AD) = 3,315/98	1998–2012	- Dementia diagnosis; AD diagnosis - DSM-IIIR or DSM-IV; NINCDS-ADRDA
Wium-Andersen et al. (2019)	Nested case-control study	58,095	61.4 (51–78)	54.2%	Never metformin-use	Ever metformin-use	Ever metformin = 14,692/2,425; Never metformin = 43,403/9,194	1995–2012	- Dementia diagnosis - ICD-10 (F00-F04, G30)
Whitmer et al. (2013)	Cohort study	14,891	NR ≥50	NR	Sulfonylureas	Metformin-use	Overall = 14,891/1,190 (metformin = 818; sulfonylurea = 372)	1999–2001	- Dementia diagnosis - NR
Scherrer et al. (2019a)	Retrospective cohort study	73,761	NR ≥50	96.8%	Sulfonylurea-use	Metformin-use	Metformin = 55,859/NR; Sulfonylurea = 17,902/NR	2000–2015	- Dementia diagnosis - ICD-9-CM
Bohlken et al. (2018)	Case-Control Study	16,552	79.7 (≥60)	43.8%	Never metformin-use	Ever metformin-use	Ever metformin = 13,134/6,265; Never metformin = 3,641/2,011	2013–2017	- Dementia diagnosis - ICD-10 (F01, F03, G30)
Secnik et al. (2020)	Prospective cohort	133,318	80.5 (NR)	50.4%	Non-use of metformin	metformin-use	Metformin = 93,130/9,023; Non-metformin = 40,188/3,261	2005–2018	- Dementia diagnosis - ICD-10 (F00-F03, G30, G31)
Sečník (2021)	Cohort study	132,402	NR (≥40)	NR	Non-use of metformin	Metformin-use	Overall (dementia) = 132,402/11,401; Overall (non-dementia) = 132,402/121,001	2007–2018	- Dementia diagnosis - ICD-10
Hsu et al. (2011)	Representative cohort study	25,393	NR (≥50)	51.1%	Non-use of antidiabetics	Metformin monotherapy	Metformin monotherapy = 1,864/66; Non-use of antidiabetics = 10,519/434	2000–2007	- Dementia diagnosis - ICD-9-CM (290.0, 290.1, 294.1,331.0–331.2 or 331.7–331.9) or abridged (A210, A222)
Liccini et al. (2016)	Cross-sectional study	198	64.9 (50–90)	NR	Non-use of metformin	Metformin-use	Metformin-use = 118/NR; Non-use of metformin = 80/NR	2014	- Cognitive impairment - RCS (<7 scores)
Tseng (2019)	Retrospective cohort study	163,405	61.8 (NR)	54.4%	Never use of metformin	Ever metformin-use	Ever metformin-use = 147,730/3,943; Never use of metformin = 15,676/713	1999–2005	- ICD-9-CM (290.0, 290.1, 290.2, 290.4, 294.1, 331.0–331.2, or 331.7–331.9) or abridged codes (A210 or A222)
Kim et al. (2019)	Cohort study	278,290	73.4 (≥60)	40.3%	Non-use of metformin monotherapy	Metformin monotherapy	Metformin-use = NR/6,698; Non-use of metformin = NR/1,905	2002–2013	- Dementia diagnosis - ICD-10
Salas et al. (2020)	Retrospective cohort study	127,178	VHA: 62.6 (≥ 50) KPW: 63.7 (≥50)	VHA:96.8% KPW: about 50%	Non-initiators metformin	Metformin Initiators	VHA: Metformin Initiators = 18,904/986; Non-initiators metformin = 93,941/6,561; KPW: Metformin Initiators = 1,793/46; Non-initiators metformin = 12,540/1,044	1996–2015	- Dementia diagnosis - ICD-9-CM
Cheng et al. (2014)	Cohort study	67,731	73.6 (≥65)	45.4%	Sulfonylureas-use	Metformin-use	Metformin-use = 1,033/NR; Sulfonylureas-use = 796/NR	2004–2009	- Dementia diagnosis - ICD-9-CM
Porter et al. (2019)	Cohort study	4,160	74.2 (≥60)	34.4%	Normoglycemia and non-use of metformin	Metformin-use with hyperglycemia	Metformin-use = 318/NR; Non-use of metformin = 3,842/NR	2008–2012	- Cognitive dysfunction - MMSE, FAB, RBANS
Moore et al. (2013)	Cohort study	1,354	73.8 (51–99)	40.5%	Non-use of metformin with T2D	Metformin-use	Metformin-use = 35/NR; Non-use of metformin = 91/NR	NR	- Cognitive impairment - MMSE (<28)
Yokoyama et al. (2015)	Cross-sectional study	1,449	68 (≥50)	64.8	Non-use of metformin	Metformin-use	NR	2012	- Cognitive impairment - MMSE (<24)
Teng et al. (2021)	Case-control study	234	67.8 (NR)	53.8%	Non-use of metformin	Metformin-use	Metformin-use = 110/35; Non-use of metformin = 361/153	2017–2019	- Cognitive impairment - MMSE
Shi et al. (2019)	Cohort study	5,528	63.2 (≥50)	97.8%	Non-use of metformin	Metformin-use	Metformin use = 2,772/NR; Non-use of metformin = 2,756/NR Total cases of dementia = 433; Total cases of AD = 71	2004–2010	- AD or dementia diagnosis; - ICD-9-CM
Ng et al. (2014)	Cross-sectional study	365	67.0 (55–93)	41%	Non-use of metformin	Metformin-use	Metformin-use (0–6yrs) = 114/15; Metformin-use (≥6 yrs) = 90/14; Non-use of metformin = 161/26	2003–2005	- Cognitive impairment - MMSE ( ≤ 23)
Akimoto et al. (2020)	Case-control study	66,085	73.8 (≥ 65)	50.5%	Glimepiride monotherapy	Metformin-use	Metformin-use = 24,090/578; Glimepiride = 4,650/142	2004–2018	- AD diagnosis - NR
Naharci et al. (2016)	Cohort study	193	75.6 (NR)	30.6%	Non-use of metformin	Metformin-use	Metformin-use = 131/27; Non-use of metformin = 62/17	NR	- Dementia diagnosis - NR
Scherrer et al. (2019b)	Cohort study	86,053	VHA: 60.8 (≥50) KPW: 63.1 (≥50)	VHA: 96.8% KPW: 50.5%	Sulfonylureas-use	Metformin-use	Metformin-use (VHA) = 56,972/NR; Non-use of metformin (VHA) = 18,215/NR; Metformin-use (KPW) = 7,546/NR; Non-use of metformin (KPW) = 3,320/NR	VHA: 1999–2015; KPW: 1996–2015	- Dementia diagnosis - ICD-9-CM
Koo et al. (2019)	Prospective cohort	732	76.7 (NR)	32.4%	Non-use of metformin	Use-metformin	Metformin monotherapy = 93/90; Non-use of metformin = 639/623	2011–2016	- Cognitive impairment - MMSE (<28) and Verbal Immediate Recall

a Available data were extracted based on mean baseline value of each included trials.

AD, Alzheimer's disease; CM, Clinical Modification; DSM, Diagnostic and Statistical Manual of Mental Disorders; FAB, Frontal Assessment Battery; ICD, International Classification of Diseases; KPW, Kaiser Permanente Washington; MMSE, Mini-Mental State Examination; N, number of patients; NR, not reported; RBANS, Repeatable Battery for Assessment of Neuropsychological Status; RCS, Rapid Cognitive Screen; T2D, type 2 diabetes; VHA, Veterans Health Affairs; yrs, years.

### Quality assessment

As shown in Table 3, the NOS score for all the cohort studies and case-control studies ranged from 7 to 9 points. The AHRQ score for each included cross-sectional study ranged from 8 to 10 points.

**Table 3:** Results of quality assessment of the included studies.

**(a) Results of quality assessment using the Newcastle-Ottawa Scale for cohort studies**.									
**Study**	**Representativeness of the exposed cohort**	**Selection of non-exposed cohort**	**Ascertainment of exposure**	**Demonstration that outcome of interest was not present at start of study**	**Comparability of cohorts on the basis of the design or analysis** ^a^	**Assessment of outcome**	**Was follow-up long enough for outcomes to occur**	**Adequacy of follow up of cohorts**	**Total**
Orkaby	⋆	⋆	⋆	⋆		⋆	⋆	⋆	7
Weinstein	⋆	⋆	⋆	⋆		⋆	⋆	⋆	7
Whitmer	⋆	⋆	⋆	⋆	⋆⋆	⋆	⋆		8
Scherrer	⋆	⋆	⋆	⋆	⋆⋆	⋆	⋆	⋆	9
Secnik	⋆	⋆	⋆	⋆	⋆⋆		⋆	⋆	8
Sečník	⋆	⋆	⋆	⋆		⋆	⋆	⋆	7
Hsu	⋆	⋆	⋆	⋆	⋆⋆	⋆	⋆		8
Tseng	⋆	⋆	⋆	⋆		⋆	⋆	⋆	7
Kim	⋆	⋆	⋆	⋆	⋆⋆		⋆	⋆	8
Salas	⋆	⋆	⋆	⋆	⋆⋆	⋆	⋆	⋆	9
Cheng	⋆	⋆	⋆	⋆	⋆⋆		⋆		7
Porter	⋆	⋆	⋆	⋆		⋆	⋆	⋆	7
Moore	⋆	⋆	⋆	⋆	⋆⋆		⋆	⋆	8
Koo	⋆	⋆	⋆	⋆	⋆⋆	⋆	⋆	⋆	9
Naharci	⋆	⋆	⋆	⋆	⋆⋆			⋆	7
Scherrer	⋆	⋆	⋆	⋆	⋆⋆	⋆		⋆	8
Shi	⋆	⋆	⋆		⋆⋆	⋆	⋆	⋆	8
**(b) Results of quality assessment using the Newcastle-Ottawa Scale for case-control studies**.									
**Study**	**Is the case definition adequate?**	**Representativeness of the cases**	**Selection of Controls**	**Definition of controls**	**Comparability of cases and controls on the basis of the design or analysis** ^a^	**Ascertainment of exposure**	**Same method of ascertainment for cases and controls**	**Non-response rate**	**Total**
Akimoto	⋆	⋆	⋆	⋆		⋆	⋆	⋆	7
Ha	⋆	⋆	⋆	⋆	⋆⋆		⋆	⋆	8
Sluggett	⋆	⋆	⋆	⋆	⋆⋆		⋆	⋆	8
Imfeld	⋆	⋆	⋆	⋆	⋆⋆	⋆	⋆		8
Hsiao	⋆	⋆	⋆	⋆	⋆⋆	⋆	⋆		8
Wium-Andersen	⋆	⋆	⋆	⋆	⋆⋆	⋆	⋆		8
Bohlken	⋆	⋆	⋆	⋆	⋆⋆	⋆	⋆	⋆	9
Teng	⋆	⋆	⋆	⋆		⋆	⋆	⋆	7
**(c) Results of quality assessment using the Agency for Healthcare Research and Quality (AHRQ) for cross-sectional studies**.											
**Study**	**Define the source of information?**	**List inclusion and exclusion criteria for exposed and unexposed subjects (cases and controls) or refer to previous publications**	**Indicate time period used for identifying patients**	**Indicate whether or not subjects were consecutive if not population-based**	**Indicate if evaluators of subjective components of study were masked to other aspects of the status of the participants**	**Describe any assessments undertaken for quality assurance purposes (e.g., test/retest of primary out come measurements)**	**Explain any patient exclusions from analysis**	**Describe how confounding was assessed and/or controlled**.	**If applicable, explain how missing data were handled in the analysis**	**Summarize patient response rates and completeness of data collection**	**Clarify what follow-up, if any, was expected and the percentage of patients for which incomplete data or follow-up was obtained**	**Total**
Yokoyama	⋆	⋆	⋆	⋆	⋆	⋆		⋆	⋆		⋆	9
Ng	⋆	⋆	⋆	⋆	⋆	⋆	⋆		⋆	⋆	⋆	10
Liccini	⋆	⋆	⋆	⋆	⋆	⋆		⋆		⋆		8

a A maximum of 2 stars can be allotted in this category, one for age, the other for other controlled factors.

#### Meta-analysis on metformin and cognitive dysfunction

**Figure 2:**
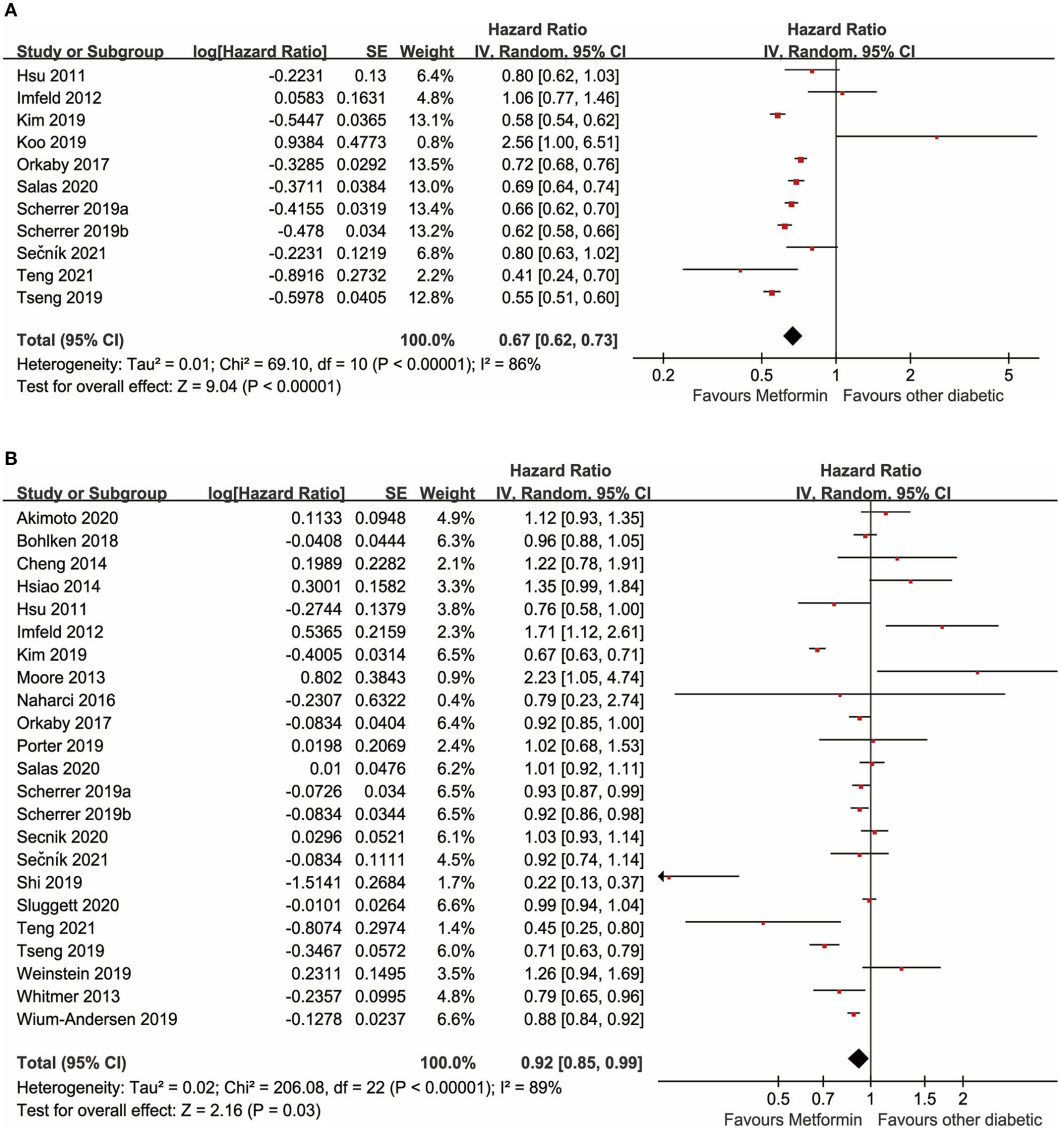
**(A)** Meta-analysis of unadjusted covariates between metformin and the occurrence of cognitive dysfunction. **(B)** Meta-analysis of adjusted covariates between metformin and the occurrence of cognitive dysfunction.

Overall, 24 studies examined the effects of metformin use on cognitive performance in patients with diabetes. Forest plots are shown in Figures 2A,B. A total of 11 unadjusted studies that could be pooled in a meta-analysis showed that diabetes with oral metformin was associated with a reduced risk of cognitive impairment (*HR*: 0.67, 95% *CI*: 0.62–0.73, *I*^2^ = 86%, *p* < 0.00001) (Figure 2A) (Hsu et al., 2011; Imfeld et al., 2012; Orkaby et al., 2017; Kim et al., 2019; Koo et al., 2019; Scherrer et al., 2019a,b; Tseng, 2019; Salas et al., 2020; Sečník, 2021; Teng et al., 2021). Similarly, the meta-analysis of 23 studies with available data revealed that metformin was associated with a reduced risk of cognitive dysfunction in adults with diabetes after adjusting for potential confounding factors (a*HR*: 0.92, 95% *CI*: 0.85–0.99, *I*^2^ = 89%, *p* < 0.00001) (Figure 2B) (Hsu et al., 2011; Imfeld et al., 2012; Moore et al., 2013; Whitmer et al., 2013; Cheng et al., 2014; Hsiao et al., 2014; Naharci et al., 2016; Orkaby et al., 2017; Bohlken et al., 2018; Kim et al., 2019; Porter et al., 2019; Scherrer et al., 2019a,b; Shi et al., 2019; Tseng, 2019; Weinstein et al., 2019; Wium-Andersen et al., 2019; Akimoto et al., 2020; Salas et al., 2020; Secnik et al., 2020; Sluggett et al., 2020; Sečník, 2021; Teng et al., 2021).

### Subgroup analysis

#### Meta-analysis on metformin and dementia

**Figure 3:**
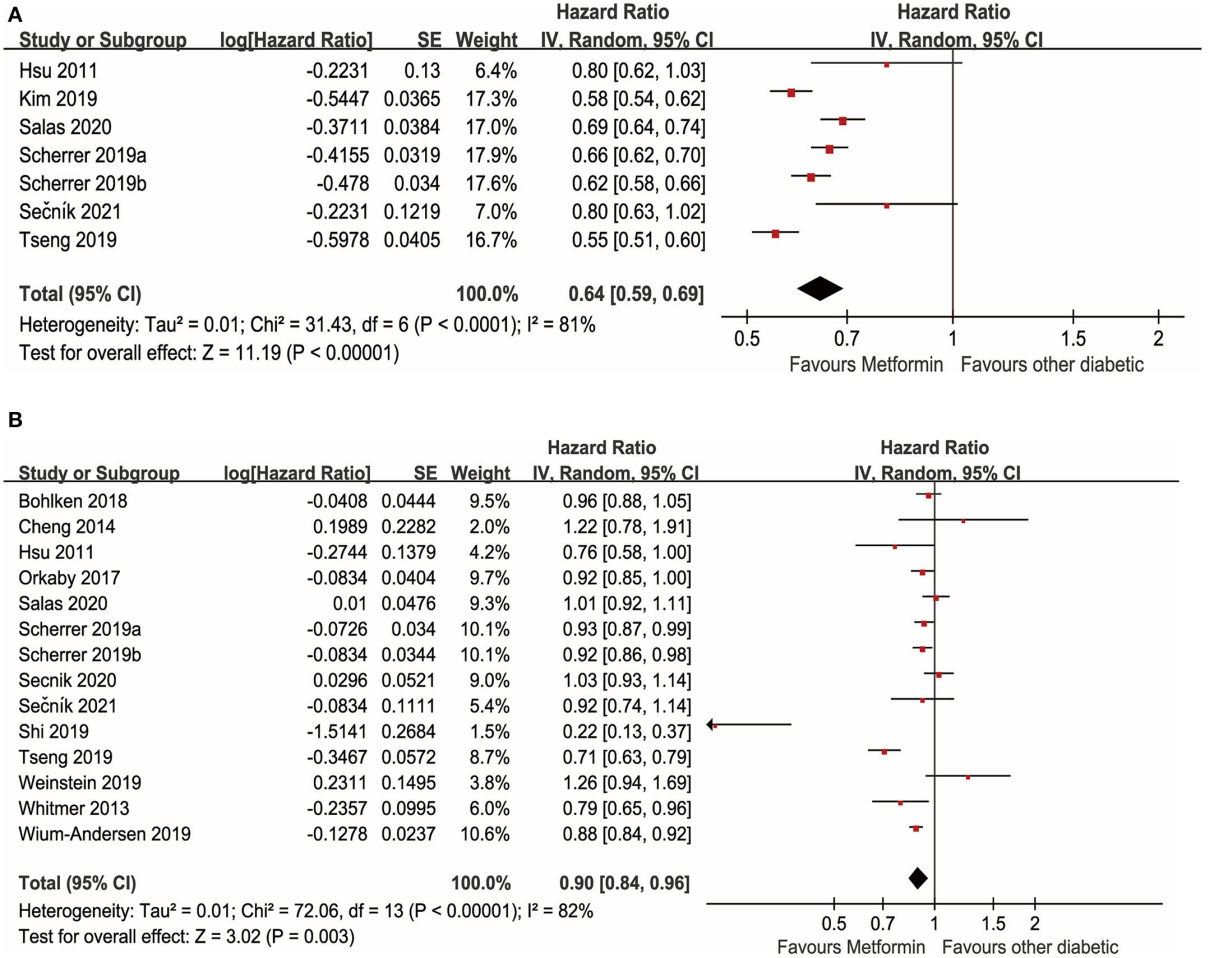
**(A)** Meta-analysis of unadjusted covariates between metformin and the occurrence of dementia. **(B)** Meta-analysis of adjusted covariates between metformin and the occurrence of dementia.

There were 15 studies with data available to examine the effect of metformin use on the incidence of dementia in adults diagnosed with diabetes. As depicted in Figure 3A, a total of 7 unadjusted studies showed that diabetes with oral metformin was associated with a decreased risk of dementia in patients with diabetes (*HR*: 0.64, 95% *CI*: 0.59–0.69, *I*^2^ = 81%, *p* < 0.0001) (Hsu et al., 2011; Kim et al., 2019; Scherrer et al., 2019a,b; Tseng, 2019; Salas et al., 2020; Sečník, 2021). Similarly, as shown in Figure 3B, a total of 14 studies with available data indicated that metformin was associated with a reduced risk of dementia after adjusting for potential confounding factors (a*HR*: 0.90, 95% *CI*: 0.84–0.96, *I*^2^ = 82%, *p* < 0.00001) (Hsu et al., 2011; Whitmer et al., 2013; Cheng et al., 2014; Orkaby et al., 2017; Bohlken et al., 2018; Scherrer et al., 2019a,b; Shi et al., 2019; Tseng, 2019; Weinstein et al., 2019; Wium-Andersen et al., 2019; Salas et al., 2020; Secnik et al., 2020; Sečník, 2021).

#### Meta-analysis on metformin and Alzheimer's disease

**Figure 4:**
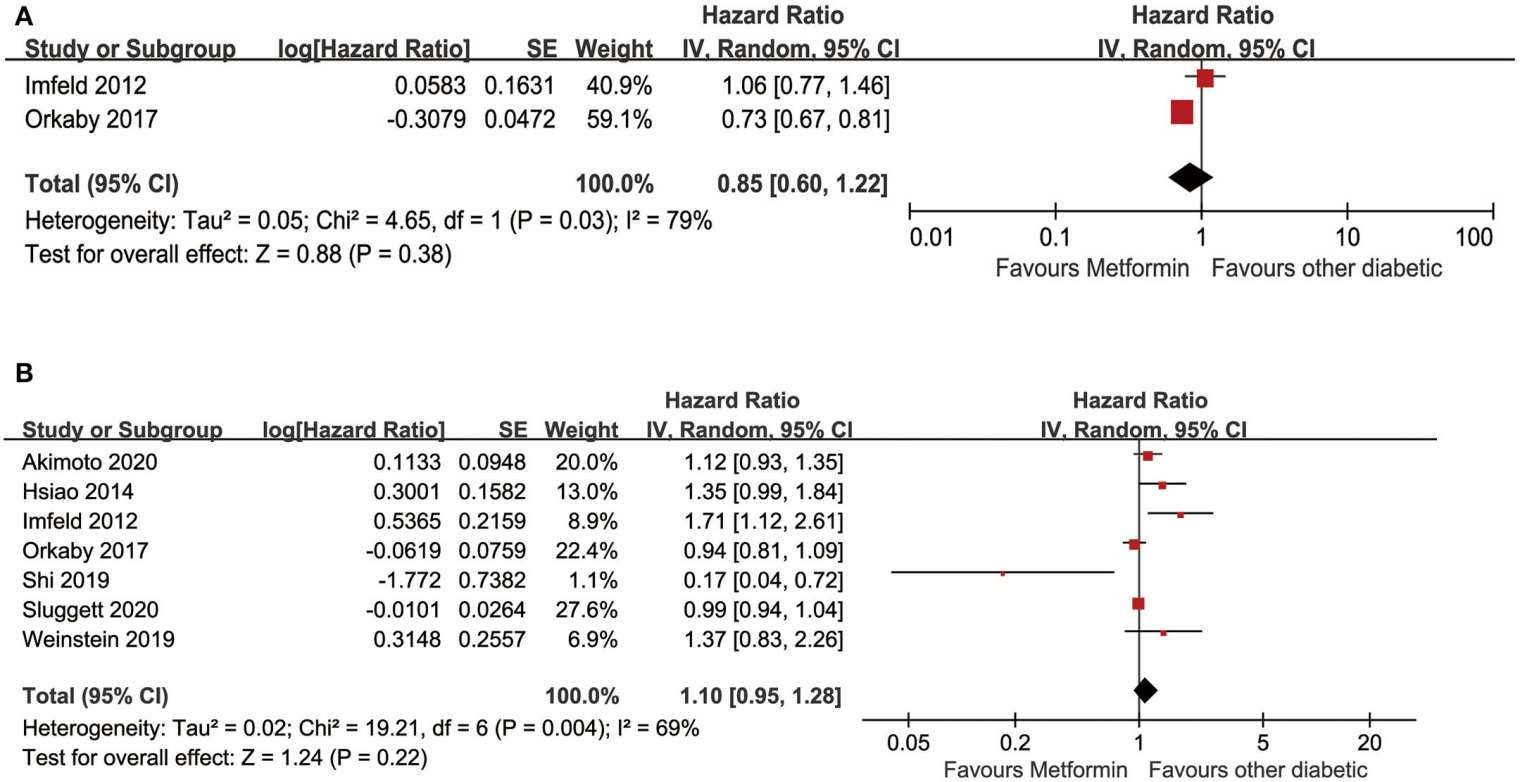
**(A)** Meta-analysis of unadjusted covariates between metformin and the occurrence of Alzheimer' s disease. **(B)** Meta-analysis of adjusted covariates between metformin and the occurrence of Alzheimer' s disease.

In the meta-analysis, seven studies with available data for evaluating the relationship between metformin use and AD in adults with diabetes were included. Forest plots are shown in Figures 4A,B. A total of two unadjusted studies showed that diabetes with oral metformin did not decrease the risk of AD (*HR*: 0.85, 95% *CI*: 0.60–1.22, *I*^2^ = 79%, *p* = 0.03) (Figure 4A) (Imfeld et al., 2012; Orkaby et al., 2017). Similarly, a total of seven studies indicated that oral metformin was not associated with a decreased occurrence of AD in individuals with diabetes after adjusting for potential confounding factors (a*HR*: 1.10, 95% *CI*: 0.95–1.28, *I*^2^ = 69%, *p* = 0.004) (Figure 4B) (Imfeld et al., 2012; Hsiao et al., 2014; Orkaby et al., 2017; Shi et al., 2019; Weinstein et al., 2019; Akimoto et al., 2020; Sluggett et al., 2020).

### Sensitivity analysis and publication bias

**Figure 5:**
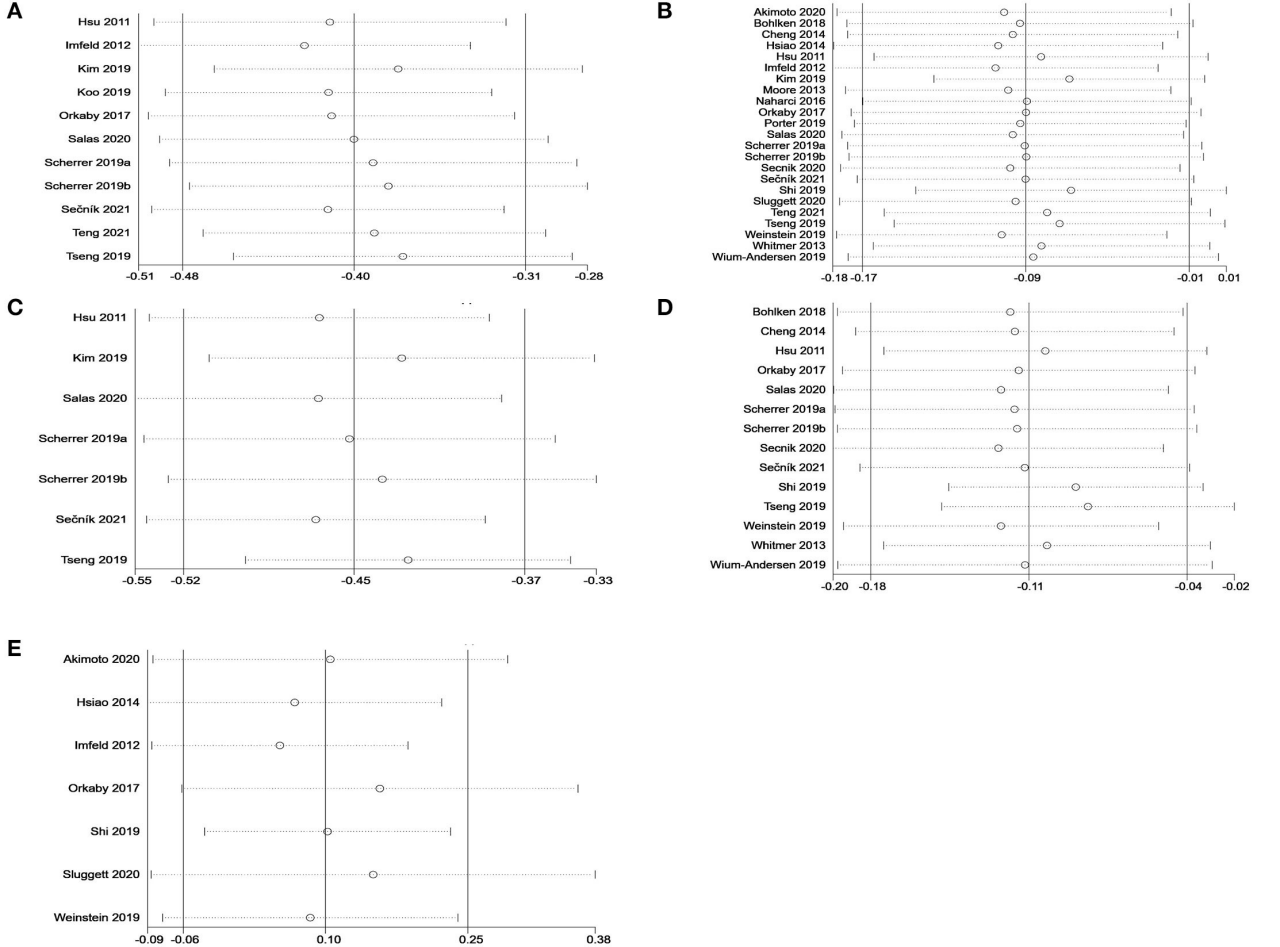
Sensitivity analysis of metformin on cognitive dysfunction in patients with diabetes in a random-effects meta-analysis of included observational studies. **(A)** All studies (unadjusted covariates); **(B)** All studies (adjusted covariates); **(C)** Subgroup meta-analysis on dementia (unadjusted covariates); **(D)** Subgroup meta-analysis on dementia (adjusted covariates); **(E)** Subgroup meta-analysis on AD (adjusted covariates).

**Figure 6:**
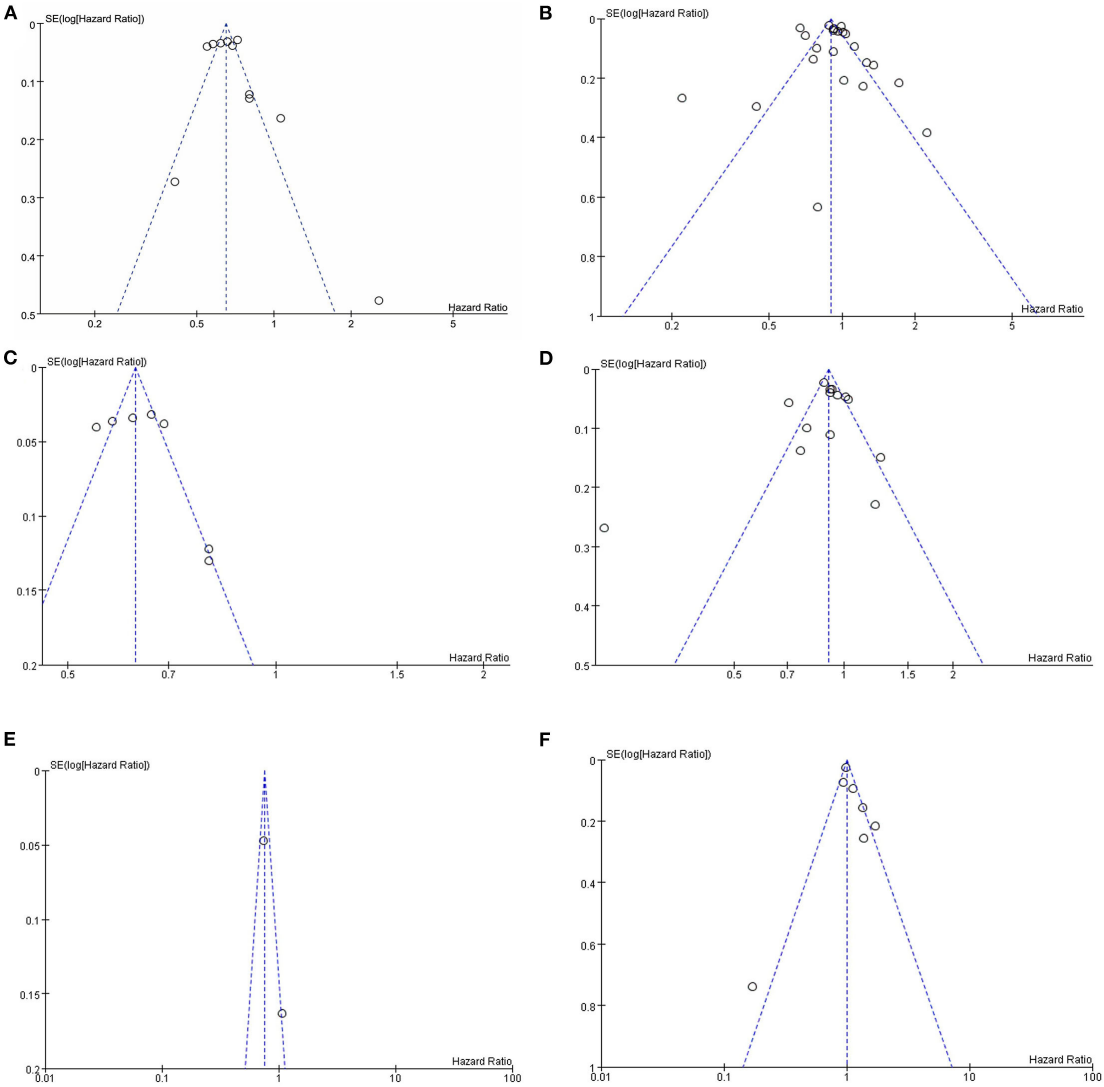
Assessment of publication bias by funnel plots of metformin on cognitive dysfunction in patients with diabetes included observational studies. **(A)** All studies (unadjusted covariates); **(B)** All studies (adjusted covariates); **(C)** Subgroup meta-analysis on dementia (unadjusted covariates); **(D)** Subgroup meta-analysis on dementia (adjusted covariates); **(E)** Subgroup meta-analysis on AD (unadjusted covariates); **(F)** Subgroup meta-analysis on AD (adjusted covariates).

Sensitivity analyses were performed to investigate the influence of each individual study on the overall meta-analysis summary estimate and the validity of the effect size. Excluding the included studies one by one demonstrated that no single study had a significant impact on the outcome of the combined analysis, suggesting that the results of this meta-analysis were stable (Figure 5). Egger's test and Begg's funnel plot did not find evidence of publication bias (*p* < 0.05) (Figure 6).

## Discussion

The aim of this systematic review and meta-analysis was to evaluate the impact of metformin on cognitive impairment in adults diagnosed with diabetes. A total of 28 observational studies met the inclusion criteria, and 24 studies were deemed appropriate in the pooled analysis for this systematic review and meta-analysis, the main findings of which included: (1) a meta-analysis reported that the protective effect of metformin therapy decreases the risk of cognitive dysfunction in patients with diabetes; (2) subgroup analyses found that oral metformin was associated with a decreased risk of dementia in patients with diabetes; (3) a subgroup analysis of meta-analysis on metformin and AD found that metformin could be associated with no significant effect on the decreased risk of AD.

Congruent with the findings that oral metformin was associated with a lower prevalence of dementia in the current study, there is some research suggesting that metformin initiation is associated with a substantially lower risk of dementia among younger African American patients (Scherrer et al., 2019a). However, the results from Salas et al. (2020) did not support initiating metformin earlier to prevent cognitive decline. The discrepancies in the results of these studies might be attributed to patient populations that differed in clinical and demographic characteristics and treatment timing. Therefore, RCTs with an optimal sample size need to be performed on the use of metformin for diabetes to confirm and extend these findings.

A subgroup analysis in the current study, based on observational studies, indicated that metformin was not significantly associated with a decreased risk of AD. In contrast, Ha et al. (2021) reported that metformin use was related to an increased risk of AD after adjusting for comorbidities and cardiometabolic risk profile by multivariable regression analyses. In addition, the treatment of diabetes with metformin cumulatively for more than 4 years significantly increased the risk of developing AD (Hsiao et al., 2014). However, Sluggett et al. (2020) showed that long-term (≥10 years) and high-dose metformin therapy had a lower risk of incidence of AD in older people with diabetes. Taken together, these results show that future RCTs with a larger sample size focusing on AD and metformin use in adults diagnosed with diabetes are warranted to explain these mixed findings.

The potential mechanisms of the relationship between metformin and cognitive performance have yet to be elucidated. Previous studies in animals have indicated that metformin could reduce cognitive impairment by reversing the harmful effects of impaired insulin signaling that causes a cascade of deleterious events, such as oxidative stress, inflammation, and tau hyper-phosphorylation (Farr et al., 2019; Gorgich et al., 2021). Moreover, the current study provides primary evidence suggesting that adults with diabetes facing a high risk of cognitive dysfunction should consider metformin as a first-line therapy.

Heterogeneity was detected in the meta-analysis of the present study. This variation could potentially be related to differences in the cumulative dose and duration of metformin, the race of the participants, and the duration and severity of diabetes. In addition, the uncertain accuracy of AD diagnoses in administrative data should be considered. Studies were included that used reliable neuropsychological cognitive assessment tools (i.e., Repeatable Battery for the Assessment of Neuropsychological Status and Frontal Assessment Battery) to report cognitive impairment instead of Minimum Mental State Examination (MMSE) in metformin users. However, the current study showed that the effect of metformin on the incidence of cognitive impairment remained effective when data were adjusted for potential confounding factors.

## Limitations

There were three main limitations in this meta-analysis. First, the findings of this review provided only very weak support for the hypothesis that metformin could prevent cognitive impairment in people without diabetes. However, the subgroup analysis of these factors could not be conducted due to the limited amount of data. Second, a subgroup analysis of studies adequately controlled for diabetes severity at baseline could not be conducted due to the limited amount of data. Finally, studies with vs. without an active comparator were not performed in the subgroup analysis in this meta-analysis.

## Conclusion

Metformin reduces the incidence of cognitive impairment but not AD in adults with diabetes. Future trials should examine the role of metformin in patients with diabetes in an RCT with a larger sample size, well-controlled confounding factors, sufficient follow-up time, and more accurate assessment of metformin exposure levels.

## Data availability statement

The original contributions presented in the study are included in the article/Supplementary material, further inquiries can be directed to the corresponding author/s.

## Author contributions

J-HZ, X-YZ, and Y-QS contributed equally to the conception of this systematic review and meta-analysis. J-HZ, X-YZ, Y-QS, R-HL, MC, and ML conceived the study design. X-YZ made search strategy and discussed with Y-QS and J-HZ. J-HZ and Y-QS were conducted the search and data collection from the included studies and performed quality assessment and data analysis. X-YZ drafted the final version of this study. All authors have approved the publication of study and take the responsibility for the integrity of the data and the accuracy of the data analysis.

## Conflict of interest

The authors declare that the research was conducted in the absence of any commercial or financial relationships that could be construed as a potential conflict of interest.

## Publisher's note

All claims expressed in this article are solely those of the authors and do not necessarily represent those of their affiliated organizations, or those of the publisher, the editors and the reviewers. Any product that may be evaluated in this article, or claim that may be made by its manufacturer, is not guaranteed or endorsed by the publisher.
