# Supplementary material for: Metformin use is associated with a reduced risk of cognitive impairment in adults with diabetes mellitus: A systematic review and meta-analysis
Source: Front Neurosci. 2022 Aug 25;16:984559. doi: 10.3389/fnins.2022.984559 (PMC9453211; doi:10.3389/fnins.2022.984559)
Supplement: Supplementary file 1 [file Table_1.docx]

| **Supplementary Table 1. Adjusted covariates** | |
| --- | --- |
|  | |
| **Study** | **Covariates adjusted for in multivariable analysis** |
| Cheng 2014 | Age, sex, hypertension, hyperlipidemia, depression and cerebrovascular disease. |
| Salas 2020 | Sociodemographic characteristics included age, race, gender, insurance type, neighborhood socioeconomic status and marital status (only available for VHA). Index year, diabetes-related (HbA1c value, HbA1c category, creatinine value), other comorbidities (obesity, hypertension, hyperlipidemia, stroke, ischemic heart disease, congestive heart failure, atrial fibrillation, traumatic brain injury, vitamin B12 deficiency), psychiatric and substance comorbidities (depression, posttraumatic stress disorder, other anxiety, bipolar disorder, schizophrenia, nicotine abuse/dependence, alcohol abuse/dependence, illicit drug abuse/dependence), other medications (statins, anticholinergic drugs, nonsteroidal anti-inflammatory drugs, antihypertensive drugs). |
| Hsu 2011 | Age, gender, type of stroke and Charlson comorbidity index score. |
| Whitmer 2014 | Age, race, education, diabetes duration, glycosylated hemoglobin. |
| Imfeld 2012 | Cases were matched 1:1 with controls with no evidence of any type of dementia or prescriptions for specific drugs to treat AD, matching was performed for age, sex, calendar time, GP, and number of years of recorded history. Adjustments were for antidiabetic drug classes, smoking, BMI, dyslipidemia, use of angiotensin-converting enzyme inhibitors and statins. |
| Orkaby 2017 | Race, sex, BMI, HbA1c, eGFR, region, coronary artery disease, heart failure, atrial fibrillation, hypertension, hyperlipidemia, peripheral artery disease, eye disease, cancer, arthritis, substance abuse, major psychiatric disease, number of drug classes, number of visits prior to baseline, year first prescription. |
| Ng 2014 | Age, gender, education, other antidiabetic medication use, fasting blood glucose, duration of diabetes, BMI, hypertension, cardiovascular illness or stroke, other medical comorbidities, eGFR, GDS, APOE ε4 allele, and duration of follow up. |
| Hsiao 2014 | Macro-, and microvascular complications, hypertension, hyperlipidemia. |
| Naharci 2016 | Age, sex, BMI, MMSE scores, and HbA1c. |
| Liccini 2016 | Age, sex, education, and HbA1c. |
| Moore 2013 | Age, sex, level of education, history of depression, serum vitamin B12. |
| Yokoyama 2015 | Age, sex, BMI, current smoker, duration of diabetes, hypertension, dyslipidemia, and  history of coronary heart disease and stroke. |
| Scherrer 2019 | Sociodemographic variables included age, sex, and marital status using the values nearest to and before metformin or sulfonylurea prescription fill. Diabetes complications (neuropathy, retinopathy, nephropathy), hypertension, hyperlipidemia, stroke or cerebrovascular disease, ischemic heart disease, congestive heart failure, atrial fibrillation or flutter, traumatic brain injury, vitamin B12 deficiency, obesity, depression, bipolar disorder, schizophrenia, posttraumatic stress disorder, and a composite anxiety disorder variable that included panic disorder, social phobia, obsessive compulsive disorder, generalized anxiety disorder, and anxiety disorder not otherwise specified alcohol abuse/dependence, illicit drug abuse/dependence, and nicotine dependence/smoking. Exposure to anticholinergic medications, nonsteroidal anti-inflammatory drugs, statins, and anti-hypertensives, neighborhood socioeconomic status, use of non-VHA care and controlled for year of prescription fill. |
| Sečník 2021 | Sex, comorbidity index, renal disease, diabetes type and duration, cardiovascular, antithrombotic, psychotropic and dementia medication, income, other antidiabetic medications, cohabitation, dementia type and MMSE. |
| Secnik 2020 | Dementia status, index year, sex, age, diabetes duration and type, Charlson comorbidity index, renal failure, cardiovascular, antithrombotic, antipsychotic, antidepressant, hypnotic/sedative and anxiolytic drugs, education, income group, usage of other antidiabetic drugs. |
| Sluggett 2020 | Region of residence, occupational social class, cardiovascular disease (stroke, hypertension, coronary artery disease, chronic heart failure, atrial fibrillation, peripheral arterial disease), psychiatric disorders (bipolar, schizophrenia, depression), renal disease, statin use, antihypertensive use, and use of sulfonylureas, insulin and other diabetes medications. |
| Teng 2021 | Age, HbA1c, hypertension, history of stroke, homocysteine, body mass index, TG and HDL-C. |
| Wium-Andersen 2019 | Multiple adjustments include age, sex, education, marital status, year of dementia diagnosis, ischemic heart disease, cerebrovascular disease, clopidogrel/warfarin/aspirin use, hypertension, obesity, hypercholesterolemia, infections, chronic obstructive pulmonary disease, inflammatory disorder, depression, alcohol use disorder, and number of acute and chronic diabetes complications. |
| Shi 2019 | Propensity score weight: balancing cohort demographics (age, sex, race), antidiabetic medication, antihypertension medication and antidyslipidaemia medication during follow-up and oral antidiabetic medication use, medical characteristics (microvascular complications macrovascular complications, hypertension, hyperglycaemia, hyperlipidaemia, renal disease, mental disease, obesity, tobacco) |
| Bohlken 2018 | Basic characteristics of study was matched (1:1) by age, sex, index year, and physician; adjusted for mean HbA1c value prior to the index date, diabetes duration, co-diagnoses, and co-therapies. |
| Porter 2019 | Age, sex, BMI, GFR, education, socioeconomic status. |
| Kim 2019 | Age, sex, comorbidities, and Charlson comorbidities index. |
| Tseng 2019 | A matched-pair cohort was created by propensity score, matching was performed for demographic data (age, sex, occupation and living region), major comorbidities (hypertension, dyslipidemia and obesity), diabetes-related complications (nephropathy, eye disease, stroke, ischemic heart disease and peripheral arterial disease), antidiabetic drugs (insulin, sulfonylureas meglitinide, acarbose, rosiglitazone and pioglitazone), commonly encountered comorbidities (chronic obstructive pulmonary disease, tobacco abuse, alcohol-related diagnoses, head injury and Parkinson’s disease) and commonly used medications in diabetes patients (angiotensin converting enzyme inhibitor/angiotensin receptor blocker, calcium channel blocker, statin, fibrate and aspirin). |
| Weinstein 2019 | Age, sex, education, eGFR, interval between exam cycles and cognitive/MRI examination (except for cognitive change outcomes), physical activity, hypertension, cardiovascular disease, stroke, total cholesterol, smoking, depression, and BMI. In a subsequent model we also controlled for HbA1c or fasting or random blood glucose (depending on cohort-specific data availability) and ApoE4. |
| Akimoto 2020 | Age, gender and common comorbidities. |
| Ha 2021 | Hypertension, ischemic heart disease, dyslipidemia, Charlson comorbidity index, diabetes complications severity index, depression, statin use, aspirin use, antiplatelet use, anticoagulant use, antihypertensive drug use, antiarrhythmic drug use, use of antidiabetic medications, fasting blood glucose levels, systolic blood pressure, diastolic blood pressure, total cholesterol levels, creatinine levels, body mass index, smoking status, alcohol consumption, and physical activity. |
| Scherrer 2019 | Sociodemographic-related (age, sex, marital status, low neighborhood socioeconomic status, VHA only insurance, medicaid/medicare, high healthcare utilization), index year, diabetes-related (diabetic nephropathy, diabetic retinopathy, diabetic neuropathy, HbA1c value, HbA1c category, creatinine value), other comorbidities (obesity, hypertension, hyperlipidemia, stroke or cerebrovascular disease, ischemic heart disease, congestive heart failure, atrial fibrillation, traumatic brain injury, vitamin B12 deficiency), psychiatric and substance comorbidities (depression, posttraumatic stress disorder, other anxiety, bipolar disorder, schizophrenia, nicotine abuse/dependence, alcohol abuse/dependence, illicit drug abuse/dependence), other medications (statins, anticholinergic drugs, nonsteroidal anti-inflammatory drugs, antihypertensive drugs). |
| Koo 2019 | Age, sex, education level, baseline cognitive function, baseline glycated hemoglobin levels, renal and liver function, BMI, hypertension, dyslipidemia, antidiabetic agents other than metformin, and baseline brain imaging abnormality. |
| Abbreviations: AD=Alzheimer’s disease; BMI=body mass index; eGFR=estimated glomerular filtration rate; GDS=Geriatric Depression Scale; GP=general practitioner; HbA1c=hemoglobin A1c; HDL-C=high density lipoprotein cholesterol; MMSE=Mini-mental State Examination; MRI= Magnetic resonance imaging; TG=triglycerides; VHA=Veterans Health Administration. | |
